# Thin and Flexible PANI/PMMA/CNF Forest Films Produced via a Two-Step Floating Catalyst Chemical Vapor Deposition

**DOI:** 10.3390/ma17235812

**Published:** 2024-11-27

**Authors:** Foteini-Maria Papadopoulou, Spyros Soulis, Aikaterini-Flora A. Trompeta, Costas A. Charitidis

**Affiliations:** Research Lab of Advanced, Composite, Nanomaterials and Nanotechnology (R-NanoLab), School of Chemical Engineering, National Technical University of Athens, 9 Heroon Polytechniou Str., Zographos, 15780 Athens, Greece; feypap@chemeng.ntua.gr (F.-M.P.); sksoulis@mail.ntua.gr (S.S.)

**Keywords:** bamboo-like CNTs, chemical vapor deposition method (CVD), CNF forests, CNF carpets, conductive thin films, electropolymerization, PANI, PMMA, vertically aligned CNFs

## Abstract

In this paper, we explore a straightforward two-step method to produce high-purity, vertically aligned multi-walled carbon nanofibres (MWCNFs) via chemical vapor deposition (CVD). Two distinct solutions are utilized for this CVD method: a catalytic solution consisting of ferrocene and acetonitrile (ACN) and a carbon source solution with camphor and ACN. The vapors of the catalytic solution inserted in the reaction chamber through external boiling result in a floating catalyst CVD approach that produces vertically aligned CNFs in a consistent manner. CNFs are grown in a conventional CVD horizontal reactor at 850 °C under atmospheric pressure and characterized by Raman spectroscopy, scanning and transmission electron microscopy (SEM and TEM), X-ray diffraction (XRD), and thermogravimetric analysis (TGA). Coating the MWCNTs with polymethyl methacrylate (PMMA) while still on the Si substrate retains the structure and results in a flexible, conductive thin film suitable for flexible electrodes. The film is 62 μm thick and stable in aqueous solutions, capable of withstanding further processing, such as electropolymerization with polyaniline, to be used for energy storage applications.

## 1. Introduction

The demand for versatile electronic devices that are flexible, rollable, wearable, and lightweight with excellent mechanical strength has led researchers to develop advanced materials for flexible electronic applications, such as energy storage devices, heat dissipation modules, and energy harvesting through thermoelectric triboelectric phenomena (e.g., in wearables) [1]. Carbon nanotubes (CNTs) and carbon nanofibers (CNFs) are increasingly explored for this purpose due to their exceptional physical, electrical, and mechanical properties [2]. CNTs are composed of concentric graphitic shells and, depending on the synthesis method, can be highly oriented (CNT forests), offering a promising solution for the development of flexible thin films that can withstand various deformations while maintaining high performance [3,4,5,6]. On the contrary, CNFs consist of conical or planar layers stacked together to yield a fiber; however, they can also form vertically aligned structures (forests, carpets) [7]. The names and descriptions of these structures are based on the definitions provided in ISO/TS 80004-3:2020(en) [8], which defines terms related to carbon allotropes in nanotechnology.

The conventional method for synthesizing CNT/CNF forests is chemical vapor deposition (CVD), which involves the deposition of a metallic catalyst layer on a substrate with specific crystallography, followed by a reduction process to activate the catalyst for CNT/CNF growth [9,10]. While CVD has proven to be a highly effective method for CNT/CNF synthesis, there are still several challenges to overcome. One of the primary difficulties is the need for a large amount of energy to sustain the high temperatures required, often between 600 °C and 1200 °C, which can make the process energy- and cost-intensive [10,11,12]. Additionally, the presence of metallic impurities, such as those from the catalyst materials used, can be a significant challenge, as these can alter the properties and performance of the produced nanomaterials, necessitating additional purification steps [13,14,15,16]. Another challenge is the difficulty in controlling the variables related to the orientation and alignment of the growing nanotubes/nanofibres. Unaligned, randomly oriented structures can limit their potential applications, particularly in areas where precise control over nanotube arrangement is crucial, such as in electronic devices or engineered composites [9,17,18].

Floating catalyst CVD (FC-CVD) methods have been developed by introducing the catalyst precursor (typically a metallocene compound such as ferrocene—Fe (C_5_H_5_)_2_) directly into the reaction chamber along with the carbon source necessary for the CNT/CNF growth. The catalyst (Fe particles), combined with the substrate’s unique crystal structure (1 0 0), enables the formation of vertically aligned, high-aspect-ratio carbon nanostructures. This method offers a simplified manufacturing approach, resulting in fewer impurities compared to techniques using pre-deposited catalysts [10,19,20]. Plasma-enhanced floating catalyst chemical vapor deposition is an even more advanced variant that takes advantage of the unique properties of microplasma to further improve the CNF growth process, enhancing the dissociation of the reactant gases, leading to higher growth rates and purity along with improved crystallinity of the nanostructures [21,22]. The synthesis of high-purity CNFs and CNF forests through this method necessitates specialized equipment and sophisticated infrastructure; thus, the acquisition of such resources presents significant challenges.

In this study, we present a simple two-step FC-CVD approach for the fabrication of highly oriented CNFs with high purity and the production of a conductive and flexible thin film, exploiting their properties. The deposition takes place in a conventional CVD system, with the catalyst and carbon source being introduced separately in the reactor, resulting in minimal impurities and lessening the need for post-treatment. The properties and morphology of the CNF forests are investigated using cyclic voltammetry (CV), thermo-gravimetric analysis (TGA), Raman spectroscopy, X-ray diffraction (XRD), and scanning and transmission electron microscopy (SEM and TEM).

## 2. Materials and Methods

### 2.1. Experimental

#### 2.1.1. CNF Forests Fabrication

**Figure 1:**
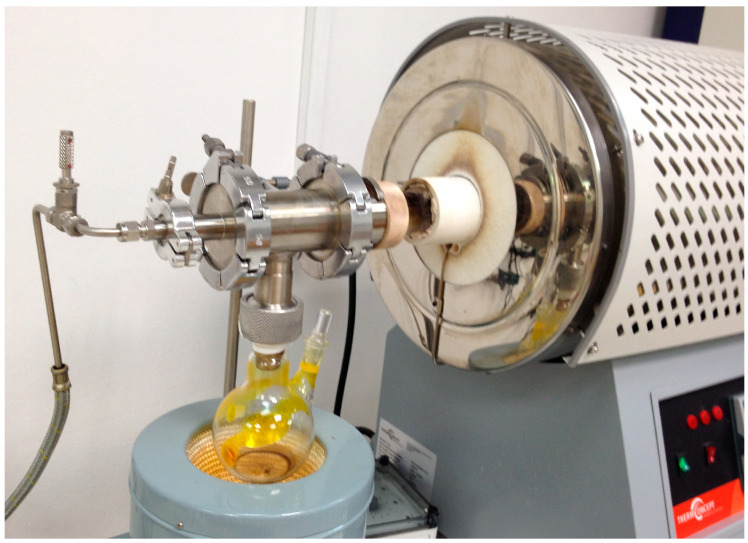
Inlet system of the CVD reactor through which the reaction solutions are introduced.

Vertically aligned CNFs were grown on a silicon wafer (P/Bor <100>, d = 100 mm, Thickness: 525 μm, Single side polished) acquired by Techline S.A., Athens, Greece, in a hot-wall CVD reactor, at atmospheric pressure. A stainless-steel tube (custom made) was used as the reactor chamber and heated through a tubular furnace that surrounds it. Argon (Ar) from a 50 L/200 bar gas bottle (Evripos Gases, Chalkida, Greece) was used as the carrier and inert gas, with a flow rate of 84 mL/min, which was the necessary flow to carry the vapors of the catalytic and carbon source solutions along the reactor chamber. The 2-step approach that was implemented refers to the two solutions used for the reaction (precursor and carbon source), which are introduced in vapor form in the reaction chamber after boiling through the inlet system presented in Figure 1. The catalytic solution (S1) is poured first, containing the iron catalytic particles coming from ferrocene (Sigma Aldrich, St. Louis, MO, USA, 8.03978) dissolved in acetonitrile (CH_3_CN, from now on ACN) (Sigma Aldrich, 34851) at different concentrations (0.05 M, 0.1 M, 0.2 M). The temperature of the heating mantel was raised above the boiling point of ferrocene (249 °C) to ensure that the vapors from the boiling solution could reach the reaction chamber and be carried away by the Ar instead of remaining within the spherical flask due to reflux. Consequently, the temperature of 253 °C was selected after trial and error. In the second step, the carbon source solution (S2) was poured into the CVD. Specifically, ACN solutions of camphor (C_10_H_16_O, Sigma Aldrich, W526606) at different concentrations (1.0 M, 2.0 M, and 3.0 M) and volumes (20, 40, and 60 mL) were tested. Similarly to S1, the selected temperature was 213 °C, above the boiling point of camphor (209 °C). The selection of ACN serves a dual role: it works as a solvent for the catalyst and as a supplementary carbon source since, as an organic solvent, it consists of carbon and, in this way, it facilitates the production of nanomaterials with minimized nitrogen content at the designated temperature [23,24]. A reaction temperature of 850 °C was employed, and the CNF forests were deposited on the silicon wafer, according to the experimental procedure described in Trompeta et al. (2016, 2019) [12,25].

#### 2.1.2. CNF/PMMA and PANI/CNF/PMMA Thin Film Preparation

The CNF forests, fabricated by the two-step method described above, were received as free-standing films using poly(methyl methacrylate) (PMMA) as a gluing matrix by casting a proper PMMA/MMA solution. This was prepared by bulk polymerization of MMA with 0.03 M of 2,2′-Azobis(2-methylpropionitrile) (AIBN, Sigma Aldrich, 11630) as the initiator. After some initial trials, it was found that the optimum casting viscosity of the PMMA/MMA solution can be achieved after polymerization in a water bath at 58 °C for 90 min (corresponding to approximately 15% polymerization yield). Polymerization yields lower than 15% resulted in a water-like PMMA coating that produced brittle films. Conversely, longer polymerization times yielded thick PMMA that could not be evenly spread across the surface, resulting in an insulating layer devoid of electrochemical activity [26].

**Figure 2:**
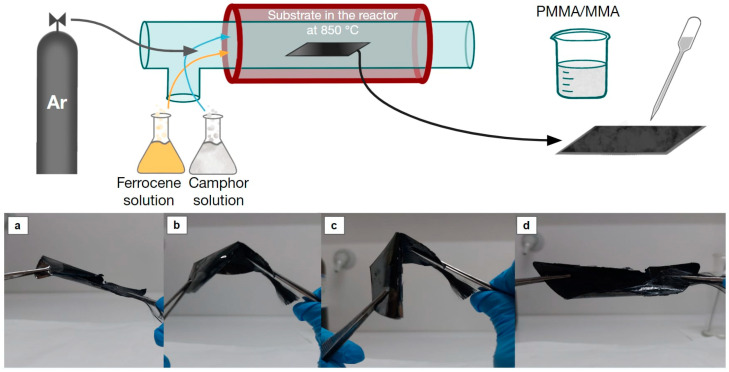
Schematic depiction of CNF forest production utilizing two solutions within a CVD reactor. Images (**a**–**d**) display the sequential bending of the same film at room temperature.

After the casting, the films were left at room temperature for a couple of hours (for the PMMA to fully solidify by the progressing polymerization reaction) and then subjected to annealing at ~80 °C for 24 h. To avoid uncontrolled warping of the films, the samples were pressed with a load of ~0.5 kg during annealing. The annealing process yielded films of uniform thickness while mitigating both curling and the development of residual stresses inherent to PMMA during cooling [27,28]. The final free-standing films were separated from the silicon wafer substrate after immersion in a concentrated alkali solution (7 M KOH) for several hours. A schematic representation of the fabrication process and the flexibility of the produced film is shown in Figure 2. To enhance the electrical properties and evaluate the stability of the fabricated thin films, a polyaniline (Aniline, Sigma Aldrich, 132934) layer was electrochemically deposited onto the surface. This polymerization was achieved via CV, utilizing an aqueous solution of 0.1 M aniline (Sigma Aldrich, 132934) with 1 M of para-toluenesulfonic acid (PTSA, Sigma Aldrich, 89866) as electrolyte.

### 2.2. Characterization Methods

The optimal volume and concentration of each solution were investigated initially by the sufficient coating of the substrate (dimensions: length 8.5–10.0 cm and average width 2.5 cm) followed by Raman spectroscopy (inVia Raman microscope equipped with a 632.8 nm laser, Renishaw, Wotton-under-Edge, UK) and TGA (NETZSCH STA 449 F5, Selb, Germany). The materials’ surface morphology was estimated via SEM using a PHILIPS Quanta Inspect (FEI Company, Hillsboro, OR, USA) microscope with a W (tungsten) filament 25 KV equipped with an EDAX GENESIS (Ametex Process and Analytical Instruments, Pittsburgh, PA, USA). The cross-section of the sample was examined by cutting a small piece from the as-received sample and placing it on the stage. Tilt mode was applied to observe the cross-section. A CM20 TEM instrument (FEI, Hillsboro, OR, USA) operating at 200 keV was utilized to identify the size and internal morphology of the carbon nanostructures. The sample was scratched from the Si wafer and grinded down in a mortar to form a fine powder. One drop of an ethanol-dispersed (0.1 mg/mL) sample was placed on a carbon-coated Cu grid and left to evaporate the solvent at RT. The crystallinity was measured with a Bruker D8 Advance Twin X-ray diffractometer (Billerica, MA, USA) equipped with a Cu K_a_ radiation source at a wavelength of 1.5418 Å. Resistance and conductivity were measured using the Keithley 4200 SCS instrument and the Ossila Four-Point Probe System (Ossila Ltd., Solpro Business Park, UK), respectively, under atmospheric pressure and ambient room temperature. SEM images were analyzed using ImageJ software (https://imagej.net/ij/, accessed on 7 September 2024) to evaluate the average length and diameter of CNF forests and CNF/PMMA thin films. 

### 2.3. Electrochemical Calculations

In order to check the electrochemical activity in different environments, the electrochemical properties and stability of the CNF forests as free-standing films were explored by CV (Potensiostat POS88, WENKING, Bank Elektronik-Intelligent Controls GmbH, Pohlheim, Germany). Different aqueous solutions were used that are representative of possible areas of applications (1.0 M PTSA as organic electrolyte, 1000 ppm NaCl as desalination solution, and 0.5 M H_2_SO_4_ as inorganic electrolyte [29]. The flexible PMMA/CNF forest films, attached to a carbon fiber approximately 4 cm in length, were tested in a three-electrode system. This system consisted of an Ag/AgCl reference electrode (RE), a platinum (Pt) wire counter electrode (CE), and the prepared thin film as the working electrode (WE). The area-specific capacitance was determined using cyclic voltammograms, with the majority of samples being scanned at a rate of 50 mV/s unless stated otherwise, and the average surface in the electrochemical cell was 5.25 cm^2^. The expression for specific capacitance [30,31,32] was:

(1) $$C_{p}\left[\frac{F}{cm^{2}}\right]=\frac{\int_{V_{1}}^{V_{2}}i\left(E\right)dE}{u\;\Delta V\;A}\;\;$$

where *C_p_* represents the area-specific capacitance, *i*(*E*) denotes the instantaneous current (A), *V*_1_ and *V*_2_ are the voltage end points (V), *u* is the scan rate (V/s), and *A* is the nominal area of the sample (cm^2^) [30,31,32].

## 3. Results

### 3.1. CNF Forests Characterization

#### 3.1.1. Morphological Characterization

**Figure 3:**
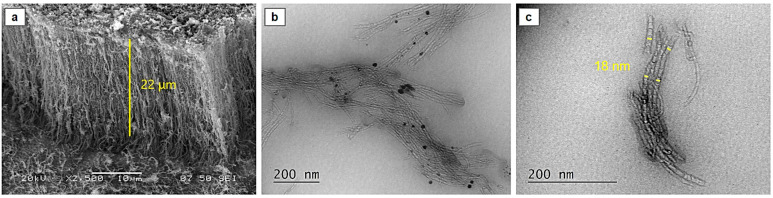
SEM image of the cross-section of the forests (**a**) and TEM image of dispersed CNFs in ethanol (**b**), measurement of tubes external diameter (**c**).

SEM analysis elucidated the morphology and length of the synthesized carbon nanostructures. The fabricated cylindrical structures exhibited a vertically aligned orientation perpendicular to the substrate (Figure 3a), with a length of approximately 22 μm. Notably, the CNT forest structures exhibited minimal amorphous carbon deposition in their as-synthesized state (Figure 3b), which is usually depicted as sphere-like structures in SEM and TEM [33]. It is evident that catalyst particles exist longwise in their structure, as indicated by black dots in Figure 3b [33]. The catalyst particles are embedded either within the cavity or on their tip, indicating a tip growth mechanism [34]. An average outer diameter of 18 nm is evident by the TEM analysis (Figure 3c). The internal structure, as revealed by Figure 3b,c, consists of consecutive stuffed cups forming continuous chains, so the produced cylindrical structures are considered CNFs [35]. It should be mentioned that in the literature, these arrays are mostly found as CNT forests or “bamboo-like CNT” instead of CNFs, despite the fact that their internal structure does not form concentric cylinders [36,37,38].

#### 3.1.2. Quality and Purity Identification

**Figure 4:**
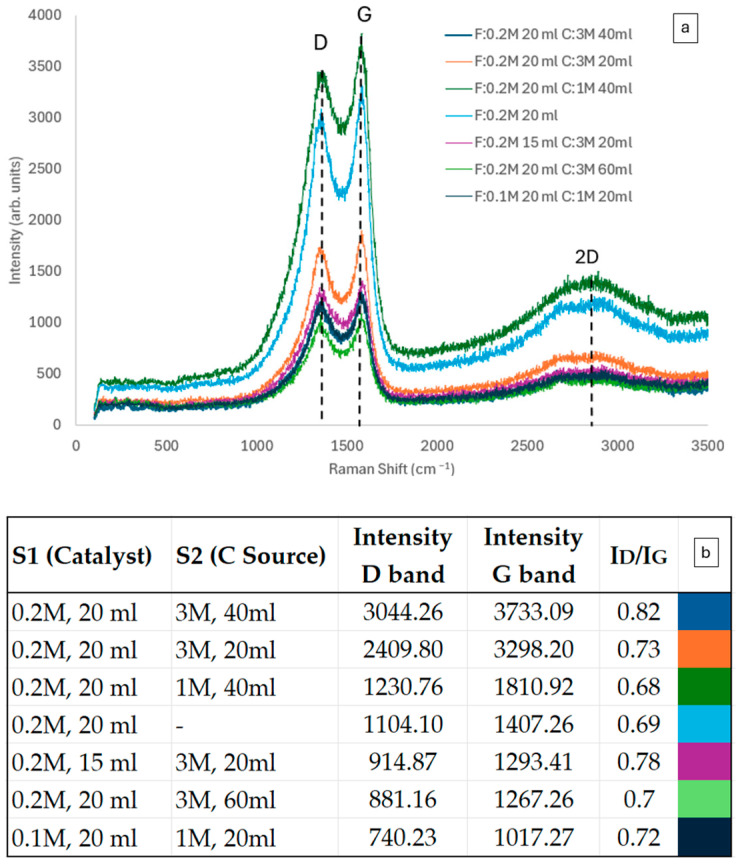
Raman spectra of CNF forests on the substrate are produced with various combinations of S1 and S2, as shown in the I_D_/I_G_ ratio stated on the bottom, with colors corresponding to those of the Raman spectra lines in Figure 4a.

Raman spectra of CNF forests synthesized under various conditions are presented in Figure 4a, with the corresponding synthesis recipes detailed in the embedded table. It is observed that all films show the characteristic peaks D (1436 cm^−1^), G (1579 cm^−1^), and G′ (2828 cm^−1^), with the D peak associated with structural disruptions and defects resulting from sp^3^ hybridization, while the G peak pertains to the tensile vibrations of sp^2^ aromatic carbon (C=C) atoms, reflecting the level of graphitization [39,40]. The presence of these peaks confirms the successful synthesis of CNFs using the two-step FC-CVD method since they are characteristic of carbon-based nanomaterials. The form of the peaks is also indicative of CNFs since they are broad and difficult to distinguish on their baseline. No radial breathing mode (RBM) peaks were evident at low wavelengths; thus, no single-walled CNTs were included in our samples [39]. 

The quality of CNF forests generated using various combinations of ferrocene and camphor solutions was assessed by analyzing the I_D_/I_G_ ratio, calculated by the height of the peaks. The characteristic Raman D-band (1360 cm^−1^) and G-band (1594 cm^−1^) were analyzed to assess the quality of the produced nanomaterials. The observed shifts in the D-band (1436 cm^−1^) and G-band (1579 cm^−1^) suggest distortions within the graphene lattice structure of the nanofibres. The G′ (2700–2695 cm^−1^) band is an overtone of the D peak involving the double phonon scattering, usually referred to as the 2D peak [39,40,41]. The calculated I_D_/I_G_ ratios (0.82, 0.73, 0.68, 0.78, 0.70, 0.69, 0.72) varied among the samples, indicating slight structural differences arising from the different compositions (Figure 4b). A lower I_D_/I_G_ ratio, indicative of a more ordered structure with fewer defects, was observed in films synthesized from solutions with higher camphor concentration (3 M) and a smaller solution feed volume (20 mL). This suggests that the camphor concentration and feedstock solution volume affect the quality of the produced CNFs [41,42]. The quantities produced using various combinations, as determined by the I_D_/I_G_ ratio from Raman analysis, indicate that the optimal composition is 20 mL of 0.2 M S1 and 20 mL of 3.0 M S2. However, it should be noted that all ratios were below 1, which is a good indication for well-structured CNFs without severe defects [39,40].

**Figure 5:**
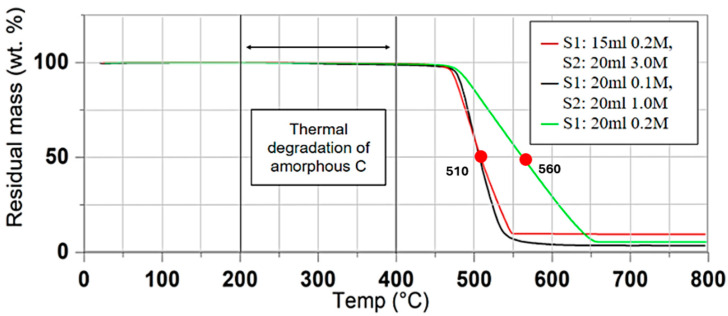
TGA for CNFs fabricated with different combinations of S1 and S2.

To investigate the influence of synthesis conditions on amorphous carbon content, as well as the thermal oxidation of the synthesized structures, samples representing the most divergent recipes were selected for the TGA. TGA was conducted under air atmosphere with a heating rate of 2.5 K/min (Figure 5). The analysis revealed a negligible mass loss up to the initial thermal degradation temperature of 476 °C, within the typical 200–600 °C range associated with amorphous carbon degradation [43]. This suggests a minimal presence of amorphous carbon in the synthesized CNFs, which is in accordance with the SEM and TEM images presented in the previous section. The samples that were grown using both solutions (catalyst solution and carbon source solution) exhibited an oxidation temperature of 510 °C. It is worth noting that the sample synthesized with 0.2 M ferrocene (green line) exhibited an increased oxidation stability (close to 600 °C) compared with the other samples. This is attributed to the excess ferrocene used in this specific recipe to maintain the consistent sample weight since only ferrocene and ACN were used for this sample (without the addition of extra carbon source) [44]. However, this sample was not rigid enough to be further processed.

The above results are in accordance with the work of Dhand et al., who developed ultrathin PVDF-CNT films with a thermal decomposition temperature of 503 °C (nitrogen used in this case for the TGA measurement) [45]. From this, it resulted that thin C-based films decomposed earlier than thicker and more dense forests. It is also common that CNFs present a lower oxidation temperature in comparison to CNTs [46]. However, it should be noted that in the study of King et al., where highly aligned arrays of super resilient single-walled CNTs were used, the oxidation temperature was 590 °C [47].

**Figure 6:**
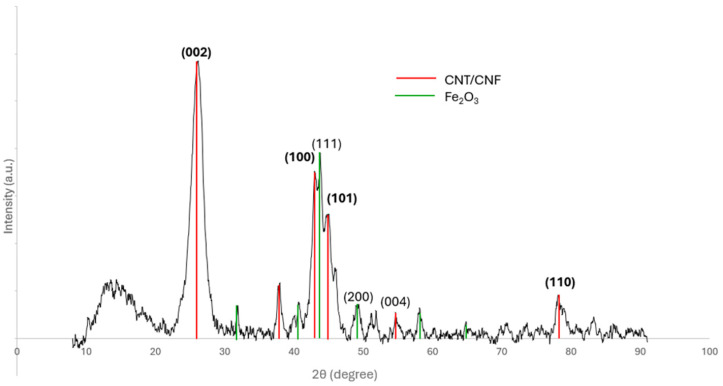
XRD spectrum of the as-produced CNF forests on the substrate.

The crystallinity of the sample with the lowest I_D_/I_G_ ratio, i.e., 0.69, was examined through XRD (Figure 6). The peaks attributed to graphite were identified: (0 0 2) at 2θ = 26°, (1 0 1) at 43.7°, and (1 0 2) at 49.3°. Additionally, peaks that correspond to iron oxides, such as Fe_2_O_3_, were evident at 38.9° [12,48]. Lastly, at angles lower than 2θ = 20°, peaks from the silicon substrate were evident. It should be noted that for aligned carbon nanostructures, according to Cao et al. [48], the intensity of the peaks is affected by the degree of the alignment, especially for the (0 0 2) peak at 26°. Its sharpness indicates that the graphite structure of the CNFs does not present significant defects [49,50].

**Figure 7:**
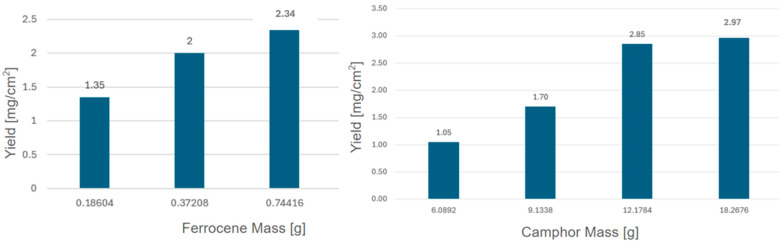
Relationship between CNF yield and ferrocene mass (**left**). Relationship between CNF yield and camphor mass (**right**). The surface area of the Si wafers used in all experiments was 20 cm^2^.

Last but not least, the relationship between CNF yield and camphor and ferrocene mass is depicted in Figure 7. The yield is expressed as the produced nanomaterial mass covering a specific surface and is measured in mg/cm^2^. The solution resulting in the highest CNF yield was the one with ferrocene 0.2 M. The highest yield of CNFs was obtained with a camphor solution at the concentration of 3M, without significant differences between the 20 mL and 40 mL solution volumes.

#### 3.1.3. Electrical Resistance and Conductivity Results 

The resistance of the produced CNF forests was noted as 81 Ω when measured on the substrate. Sheet resistivity and conductivity measurements yielded values of 47 Ω/square and 950 to 966 S/m, respectively. While these resistance measurements are significantly lower than the 4 to 498 kΩ range reported for MWCNTs in the literature, the measured conductivity values are lower than typically observed for similar structures [51,52,53]. This discrepancy could be attributed to the minimal concentration of functional groups present in the synthesized CNFs and the lack of further thermal treatment.

### 3.2. CNF/PMMA Thin Film Characterization Results

#### 3.2.1. Thin Film Morphological Analysis

**Figure 8:**
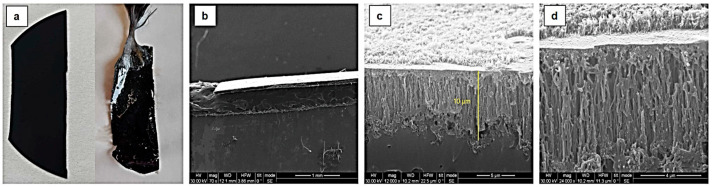
SEM image of the produced free-standing CNF forest thin film. (**a**) CNF forest as produced and grown on the Si wafer and after the formation of the stand-alone film with PMMA, (**b**) SEM image of the lateral view of the CNF forest, (**c**) thickness measurement of the CNF forest, (**d**) magnification of the cross-section.

Following the synthesis of vertically aligned CNF forests on a Si substrate and their subsequent separation from the silicon substrate, as described in Section 2.1.2, the resulting films exhibited a smooth surface morphology and a thickness of approximately 10 μm (Figure 8). A significant reduction in film thickness was observed after the application of the PMMA coating. This reduction can likely be attributed to the volumetric contraction of the MMA/PMMA solution as it polymerized and solidified around the CNFs.

#### 3.2.2. CNF/PMMA and PANI/PMMA/CNF Forests Thin Film Raman Spectra

**Figure 9:**
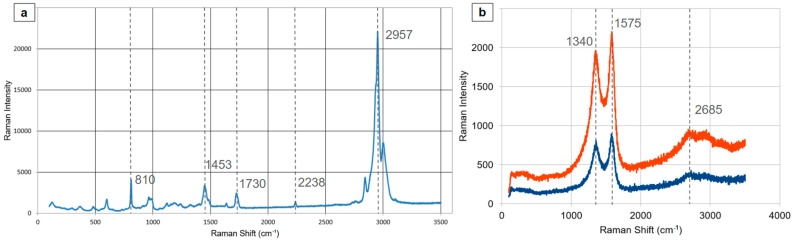
(**a**) Raman spectra of CNF/PMMA thin film presenting mainly the characteristic peaks of PMMA, (**b**) comparative diagram of Raman peaks of CNF forests on the substrate (orange) and PANI/PMMA/CNF forest film (blue).

Raman spectroscopic analysis was conducted on free-standing films incorporating PMMA (Figure 9a). Samples displayed characteristic Raman spectra for PMMA; notably, the distinctive PMMA peak at 1453.24 cm^−1^ is situated between the expected PMMA peak at 1460 cm^−1^ and the D-band of CNFs at 1436.4 cm^−1^. This shift is likely attributed to π-π interactions occurring at the nanofiber surface [54]. Further measurements on films subjected to aniline electropolymerization, resulting in a multi-layered structure of carbon nanotubes, PMMA, and PANI, revealed the presence of only the characteristic peaks of MWCNTs and PANI at 1340, 1575, and 2685 cm^−1^ (Figure 9b) [55].

#### 3.2.3. Electrical Resistance and Conductivity Results of PMMA/CNF Forest Films

The fabricated PMMA/CNF forest thin films exhibited a sheet resistance of 10 Ω/square. Resistivity measurements yielded a value of 90 μΩ·m, corresponding to a conductivity ranging from 9.3 to 11 kS/m. These conductivity and resistivity values are notably higher than those reported in the literature for similar structures incorporating PMMA and unmodified CNTs at room temperature [26,53,56]. This discrepancy could be attributed to the shrinkage of the PMMA matrix during fabrication. This shrinkage may force the initially dispersed CNTs into closer proximity, promoting the formation of a conductive network within the polymer matrix and thereby enhancing overall conductivity [26,57].

#### 3.2.4. Cyclic Voltammetry and Electropolymerization of PANI

**Figure 10:**
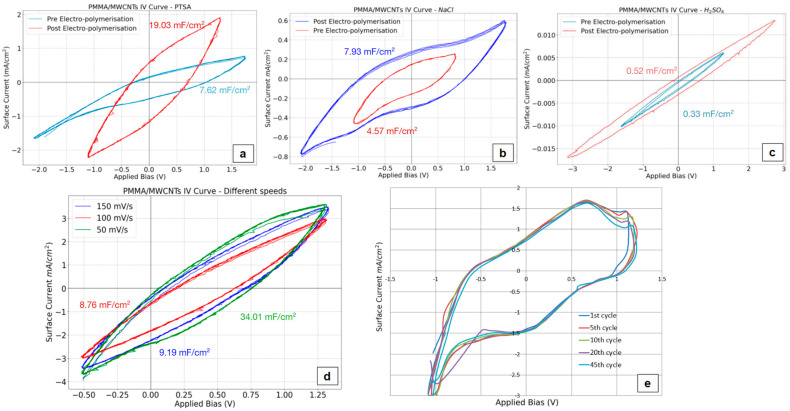
Cyclic voltammograms on CNF/PMMA thin films under different conditions. The area-specific capacitance increases after the electropolymerization of PANI in different solutions (**a**–**c**) is inversely proportional to the scan rate (**d**) and remains the same after 45 consecutive cycles (**e**) repetitive scans in a 1.0 M PTSA solution.

Cyclic voltammetry serves a dual purpose: it not only measures stored charge but can also facilitate the electropolymerization of polyaniline onto free-standing films. This process aims to enhance the film’s charge storage capacity while simultaneously affording additional protection to the nanofibers, as electropolymerization preferentially occurs on the more exposed side. Analyzing the active current in an aqueous PTSA solution revealed that electropolymerizing the film with polyaniline, a conductive polymer, significantly impacted the electrochemical behavior. Specifically, the oxidation potential dropped from 2.1 V to 1.3 V, while the reduction potential shifted from −1.6 V to 1 V (Figure 10a). Attempts to measure current in organic solutions proved unsuccessful due to the PMMA film dissolving in these solvents.

Further investigation using cyclic voltammograms demonstrated that electropolymerization with polyaniline (in a 1 M PTSA aqueous solution) led to an increase in active current, resulting in the area-specific capacitance shifting from 7.62 mF/cm^2^ to 19.03 mF/cm^2^. The layering of PANI enabled the film to store charge across a wider voltage range, spanning from −2 V to 1.7 V in 1 M NaCl solution (Figure 10b), albeit at the cost of a lower active current in those conditions (4.57 mF/cm^2^ to 7.93 mF/cm^2^ after the electropolymerization). The stability of uncoated films was also assessed by repetitive scanning in the region [−1.0 V, +1.2 V] (scan rate 100 mV/s) in a 1.0 M PTSA solution, and the measured CVs are almost the same for at least 45 repetitive scans (Figure 10e). To evaluate the film’s suitability for lithium battery applications, measurements were conducted in a 0.5 M H_2_SO_4_ solution (Figure 10c), simulating a lithium battery environment. However, the low active current values obtained suggest that these films are not well-suited for lithium or organic batteries. Finally, the influence of scan rate on the films’ charge storage capacity was examined (Figure 10d). Increasing the scan rate in a 1.0 M PTSA solution from 50 to 100 and 150 mV/s resulted in a decrease in c_p_ 34.01 to 8.76 and 9.19 mF/cm^2^, respectively. This phenomenon is likely attributed to the inherent lag in the materials’ response to rapid changes in the current direction [46]. 

## 4. Discussion

A novel and straightforward method for the reproducible synthesis of vertically aligned carbon nanofibres using a conventional CVD furnace has been investigated. This method is based on the vaporization of two separate solutions: a catalytic metalorganic solution and a carbon source organic solution, leading to a floating catalyst approach, where they react at 850 °C on the substrate. Following the synthesis of the CNF forests is their isolation from the substrate. To achieve this, a PMMA solution is applied to a CNF-covered substrate and polymerized under pressure to create a uniform, thin, flexible, and conductive film. Characterization methods demonstrated the production of high-purity CNFs with an I_D_/I_G_ ratio comparable to that achieved by other thin film deposition techniques, such as spray pyrolysis [16] and specialized CVD methods like PECVD, without requiring further purification processes [19,58,59]. The resulting thin films are flexible, conductive, and stable, suitable for additional processes like electropolymerization. A higher I_D_/I_G_ ratio indicates a higher defect degree, with factors like the nanofibre’s aspect ratio (length-to-diameter ratio) and surface modifications (e.g., crosslinking, functionalization) contributing to defect formation [60,61]. Lower aspect ratios typically lead to more defects, negatively impacting the CNFs’ mechanical and electrical properties [62]. Electropolymerization of PANI on PMMA/CNF forest films results in area-specific capacitance comparable to recent literature in the field [22,31,32,63]. With this technique, it is possible to produce doped carbon nanostructures that, due to the reduced need for further processing, can maintain their oriented structure and the unique characteristics that this structure imparts [64]. Further study of the growth mechanism of carbon nanofibres with floating catalysts and the morphological and electrical characteristics of the produced membrane is necessary. However, the measurements and characterizations presented constitute encouraging results for the continuation of the method, with the most suitable application being flexible electrodes for sensors [5,65,66,67].

## 5. Conclusions

This study employed a simple two-step floating catalyst chemical vapor deposition method to fabricate highly oriented, high-purity bamboo-like carbon nanotube forests that belong to the CNF carbon allotrope category for conductive and flexible thin film applications. The decoupled introduction of the catalyst and carbon source in a conventional CVD system minimized impurities, reducing the need for post-treatment processes. SEM revealed vertically aligned CNFs perpendicular to the substrate. TEM analysis confirmed the formation of CNFs with an average outer diameter of 18 nm. The as-synthesized CNF forests exhibited minimal amorphous carbon deposition, with I_D_/I_G_ ratios ranging from 0.68 to 0.82 depending on the precursor composition. The CNF forests exhibited a sheet resistance of 47 Ω/square and a conductivity ranging from 950 to 966 S/m. Incorporation of the CNF forests into a PMMA matrix yielded thin films with a sheet resistance of 10 Ω/square, a resistivity of 90 μΩ.m, and a conductivity ranging from 9.3 to 11 kS/m. The PMMA coating reduced the film thickness to 10 μm. Subsequent electropolymerization of PANI on the PMMA/CNF films increased the area-specific capacitance from 7.62 mF/cm^2^ to 19.03 mF/cm^2^ while maintaining stability in an aqueous environment.

## Data Availability

The raw data supporting the conclusions of this article will be made available by the authors on request.
